# Promoting physical activity and youth development in schools: the case for near-peer coaches

**DOI:** 10.3389/fpubh.2024.1345282

**Published:** 2024-03-08

**Authors:** Christine St. Pierre, Jerita Mitchell, Win Guan, Jennifer M. Sacheck

**Affiliations:** ^1^Department of Exercise and Nutrition Sciences, The George Washington University Milken Institute School of Public Health, Washington, DC, United States; ^2^Up2Us Sports, New Orleans, LA, United States; ^3^Social Insights Research, LLC, New Orleans, LA, United States

**Keywords:** physical activity, school environment, children and youth, near-peer coaching, sports-based youth development

## Abstract

**Background:**

Sports-based youth development (SBYD) programs provide an inclusive, supportive environment for promoting physical activity as well as nurturing the development of life skills which, in combination, promote physical, mental, and emotional health in youth. The Up2Us Sports SBYD program was implemented in six schools in New Orleans, Louisiana in 2020–2022, where near-peer coaches from the community were placed in schools and present throughout the school day. The intervention period straddled the COVID-19 pandemic as well as extreme weather events, modifying program delivery.

**Process/methods:**

An exploratory case study was conducted to understand participant experience amid program disruptions and modifications, as well as their perceptions of program impact on physical activity and health. Interviews with coaches (*n* = 7), focus groups with youth (*n* = 14) and program observation data were triangulated to provide a description of the case.

**Results:**

The major theme that emerged from the case study was the centrality of the near-peer mentorship relationships between coaches and youth. Participants believed near-peer relationships facilitated life skill development and increased opportunity for physical activity in schools, but pressures on coaches’ time and external challenges in the community were limiting factors to the extent of program impact.

**Conclusion:**

This community case study demonstrates the potential role for near-peer mentors in influencing the health and wellbeing of youth from under-resourced communities and highlights the opportunity for school-based SBYD programming to provide youth with a consistent source of both relational and physical activity support.

## 1 Introduction

According to physical activity surveillance data from 120 countries, more than three-quarters of the global youth population do not engage in the recommended 60 min per day of moderate to vigorous physical activity (1). Low levels of physical activity among children and youth are associated with poor physical (2, 3) and mental (4, 5) health outcomes throughout the life course, and childhood physical activity habits are likely continue into adulthood (6, 7). Amidst these already existing concerns, the disruptions and isolation of the COVID-19 pandemic greatly exacerbated worrying trends in the mental health of children and youth, prompting the U.S. Surgeon General to put out multiple advisories on the issue (8, 9) and make protecting youth mental health a key priority. Given the associations between higher youth physical activity and a range of physical (10), mental (11), and social–emotional (12) health benefits, there is great opportunity for addressing multiple youth health concerns synergistically through strategies that promote both physical activity and psychosocial development.

Sports-based youth development (SBYD) programs offer a strategy to increase physical activity levels in youth while also cultivating the development of social and life skills (13), in turn promoting multiple positive health outcomes. SBYD programs have been shown to increase perceived athletic competence among youth (14), and higher perceived competence has been associated with higher levels of physical activity (15). These programs have also demonstrated an effect on the development of life skills that foster overall health and wellbeing, such as self-worth, impulse control, and social competence, by intentionally teaching these skills as a part of programming (16, 17). Central components of SBYD programs include surrounding youth with protective factors, such as motivating, inclusive, and safe environments, and mentorship relationships with adult coaches who have been trained in youth development and in building trust and connection (18, 19). The youth-coach relationships have been identified as a facilitator of life skill development (19), but there is also an identified need for more coaches from the local community in SBYD programs (20). Furthermore, there is a call for greater collaboration between SBYD programs and other community entities to go beyond a focus on individual outcomes and leverage resources to build health-promoting environments (21).

The present study reports on the Up2Us Sports SBYD program, which addresses both limitations identified above: program coaches are intentionally recruited from the communities they serve, and program delivery is integrated into the school-day of partner schools to build a healthier school environment. The coaches in this study are young adult “near-peers” from the same or similar communities and demographic backgrounds as the youth they serve. This similarity in background and proximity in age provides youth with mentors who understand their context and experience (20). Additionally, school-based SBYD coaches offer programming universally to all enrolled students and are thus well-poised for maximal reach and impact on positive youth outcomes (22). We therefore used an exploratory case study approach (23) to investigate coach and youth experiences with the Up2Us Sports SBYD programs implemented in New Orleans, Louisiana schools. Our objective was to understand how SBYD coaches and youth perceived program implementation and impact on physical activity and broader health outcomes.

## 2 Context

Up2Us Sports is a national service program that has trained over 3,700 coach-mentors since 2010 through their Up2Us Coach flagship program. These coaches in turn have served more than 655,000 youth from historically under-resourced, largely urban communities through partnerships with recreation departments, non-profits, and local schools. Up2Us Sports provides extensive training for their coaches on creating an inclusive environment and building strong mentoring relationships with youth from all backgrounds and fitness levels. A key feature of the Up2Us Coach program is that the majority of coaches are near-peers recruited from the communities in which the program is implemented. Among Up2Us Sports large city program sites, New Orleans, Louisiana, is unique in that the coaches are primarily school-based, as opposed to working with community organizations or recreation departments that offer only out-of-school programs.

New Orleans is highly geographically segregated by race, and life expectancy has been found to differ by as much as 25 years between city ZIP codes with the highest and lowest life expectancy (24). In New Orleans schools, 92% of pupils are students of color and 80% are economically disadvantaged (25)—representing populations at greater risk for experiencing health disparities. Furthermore, youth in Louisiana are more likely to not be active for 60 min a day compared with U.S. youth as a whole (26). With many existing challenges to multiple aspects of youth health, including but not limited to low physical activity, New Orleans provides an important context in which to investigate the effectiveness of the SBYD approach in increasing physical activity and facilitating positive youth health outcomes.

## 3 Key programmatic elements and events affecting implementation

The present study is part of a wider project, Creating Opportunities for Adolescents through Coaching, Healthy Eating, and Sports (COACHES), which faced multiple external barriers to implementation, including the COVID-19 pandemic and major adverse weather events. The COACHES Project was initially conceived to evaluate the impact of school-based Up2Us Coach programs on physical activity and physical fitness, social–emotional learning, and nutrition among New Orleans middle school students. Coaches were recruited into the Up2Us Coach program from local communities to deliver free, inclusive SBYD programming at school sites. In addition to receiving the full Up2Us training, coaches participating in the project would take part in additional, contextualized training sessions on youth nutrition and physical activity. Coaches were recruited and began Up2Us training in the Fall of 2019. Baseline data for the COACHES project were collected from three intervention and two comparison schools in January 2020, SBYD programming began in the intervention schools, and repeated measurement data collection was planned for two points during the 2020–2021 school year.

Each of the school years over which the COACHES study was implemented, however, was severely disrupted, primarily by the COVID-19 pandemic, but also by multiple large weather events. School closures and transitions between virtual, hybrid, and in-person learning models in 2020 and 2021 prevented the full implementation of the COACHES project as planned. The project was extended into the 2021–2022 school year, but Hurricane Ida, which struck in August 2021, displaced families, significantly damaged multiple schools, and again altered learning models and programming. Despite these unexpected events, coaches were still trained and placed in schools and were able to offer SBYD programming during virtual learning, as well as through in-person physical education classes, elective courses, and after school activities. Additionally, evidence-based (27) coach trainings focused on nutrition and physical activity were developed and provided as planned. Youth engaged in the programming and still participated in modified data collection, providing early insights into their experiences during the pandemic (28). At the end of the COACHES study period in Spring 2022, we undertook the qualitative study described here to investigate the participant experience amidst the programming disruptions and modifications.

## 4 Methods

### 4.1 Study design

We chose an exploratory case study design to allow us to consider the program within its unique context using multiple data sources (23) and understand details of the program through the perspective of participants (29). With the many unusual circumstances surrounding program delivery, a case study methodology is an appropriate approach for an exploratory investigation into participant experiences during this period (23). The case study methods included interviews and focus groups with program coaches and youth participants, program observation, and informal interviews with Up2Us program staff. The rationale for these multiple methodologies was to allow for triangulation of data to increase validity through verification of themes emerging the analysis (30). The research was approved by the George Washington University Committee on Human Research Institutional Review Board.

### 4.2 Participants and recruitment

All active Up2Us school-based coaches in New Orleans (*n* = 11) in Spring 2022 were invited to participate in the study to include as many perspectives as possible in the case description. Coaches were contacted by email to explain the study, and their participation was requested on a voluntary basis. Originally, eight coaches at a total of four schools agreed to participate. In the wake of the pandemic, outside access to schools was limited, and coaches, who were embedded in the schools, served as the primary facilitators of contact with school administrators. The schools available for youth focus group recruitment were thus limited to a convenience sample of the four schools with participating coaches. A research team member provided administrators at these schools with information about the objectives for this study and requested their participation. Three of the schools agreed to participate, and the coaches assisted in recruiting a convenience sample of up to 10 youth from their school for a focus group. Coaches provided informed consent, and parental consent and child assent were obtained for youth participants. Coaches received a $50 stipend and youth received a $25 gift card as tokens of appreciation for their participation.

### 4.3 Data collection

Interviews and focus groups were conducted by a trained PhD student and a trained undergraduate student under the supervision of a senior university faculty researcher. None of the researchers were previously known to the participants. In schools where there were multiple coaches (*n* = 2), group interviews were conducted. The group interview approach allowed for interactions between coaches during the discussion, providing depth of inquiry that may not arise from individual interviews (31). The facilitators used semi-structured discussion guides developed via discussion between all study authors. The guides contained open-ended questions for exploring participant perceptions of (1) their program experience, and (2) connections between program experience and their physical activity and health. Facilitators asked probing questions and engaged in member checks to support confirmability of participant perspectives. All interviews and focus groups were held at schools in a quiet room or outdoor space and were audio recorded.

To further inform the case description and build context for the interview and focus group responses, facilitators observed programming at two sites and had informal interviews with program staff and coaches at all four sites. These observations and conversations provided additional insight into nuances in program implementation and school culture across the different sites. The facilitators also attended a meeting with representatives from several programs engaged in youth development work in New Orleans, which provided additional data on the ongoing coordination and partnerships to support youth across the city. The facilitators took detailed field notes during these observations, informal interviews, and meetings or as soon as possible after their conclusion. The field notes were then incorporated into data analysis alongside the focus group and interview transcripts.

### 4.4 Data analysis

All interviews and focus groups audio recordings were transcribed verbatim using NVivo Transcription software (QSR International). A research team member reviewed the audio recordings and transcripts together for accuracy and removed any identifying information. Cleaned transcripts were coded and analyzed using NVivo 12 (QSR International) We developed an initial codebook based on the discussion guides focused on identifying salient elements of participant experiences and connections to physical activity and health. The same two coders reviewed each transcript and the field notes multiple times, using iterative inductive analysis to identify patterns in the data, add codes as needed, and distill the codes into themes. The coders met after each round of transcript review to discuss patterns and update codes as needed. Differences in coding were discussed until consensus was reached. One overarching theme with three subthemes emerged from the analysis.

During data collection in Spring 2022, a number of events outside the control of the research team affected the richness of the data we were able to collect. First, the only consenting female coach was unable to participate in an interview. Secondly, unforeseen changes to the school schedule curtailed the length of two of the youth focus groups. These methodological constraints affected our ability to collect data from a fully representative participant sample. In our results and discussion, therefore, we focus on a description of and reflection on the local participant experience, which nevertheless provides lessons that can inform future programming.

## 5 Results

Seven coaches (100% male, 100% Black, 24.4 ± 5.1 years) at 4 schools participated in the interviews, and focus groups were held at 3 schools with a total of 14 students (71% male, 93% Black, 14.8 ± 1.7 years), for a total of 21 participants. Demographic data for coaches and youth are displayed in Table 1.

**Table 1:** Participant demographic characteristics.

Variable	Coaches (*n* = 7)	Youth (*n* = 14)
Age (y ± SD)	24.4 (5.1)	14.8 (1.7)
Race (%)		
African-American/Black	100%	92.9%
Caucasian/White		7.1%
Ethnicity		
Hispanic/Latino	14.3%	14.3%
Sex		
Male	100%	71.4%

The centrality of the near-peer relationships between coaches and youth to the program experience was the major theme identified through the triangulation of coach interviews, youth focus groups, and program observation. Across every interview and focus group, coaches and youth noted ways coaches were relatable and “more like the youth” than other adults in the school specifically because they were closer in age and from similar backgrounds. One youth participant described it this way:

“I feel like they [the coach] might know more because they are also younger and they might have experienced it and their brain might be able to go quickly to it and might be able to understand like what we are saying.”—*Participant 14.*

Under this broader theme, we identified three subthemes: the impact of near-peer relationships beyond programming time, increased opportunity for physical activity facilitated by near-peer relationships, and limitations to the impact of the near-peer relationships.

### 5.1 The impact of near-peer relationships beyond programming

Although SBYD programming facilitated the initial opportunity for near-peer mentor relationships to form, both coaches and youth described the impact of these relationships extending to multiple areas of life. In a particularly tight-knit group at one of the schools, participants talked about how the strength of the bond with their coach motivated them to support one another in making positive choices. As examples, youth discussed how their relationship with their coach influenced their decisions on matters that could have a major impact on their life trajectory, including staying in school.

“And it also made me look different at school, because last year, I wasn’t coming, I was about to drop out. But when I met [Coach], he was like, stick to it, you know.”—*Participant 11.*

“We try to keep each other out of trouble, you know, prevent all that because you know, that would look bad on our group. We try to really look good for [Coach], like we try to look as best we could, so we try to hold each other accountable if something going wrong, try to talk to each other.”—*Participant 8.*

Youth described additional ways their relationships with their coaches affected them beyond programming time, including striving for accomplishments in multiple areas and also in personal growth. They invited their coaches to significant events, such as band performances or school awards, noting their coaches were important supporters in their lives. Youth also described taking risks in opening up to others, an action they attributed to the bonds they built in the program.

“One of my favorite memories was [learning drums] with [Coach]. Like me and him are like, really close…he really inspired me to like push myself.”—*Participant 14.*

“I like being around a group of people that I can really express myself to, and I do not really…I cannot really express myself to a lot of other people.”—*Participant 12.*

Coaches shared a similar perspective on the role of the mentorship relationship in influencing youth choices and reactions in various challenging situations. In their roles, they are on site through much of the school day, and in each interview, coaches shared examples of opportunities both during and outside of programming time to mentor youth in navigating challenges they were facing. They related examples of conversations with youth where they consciously decided against illegal means of making money that were readily available to them or turning to fighting when they were upset with someone.

“They will come tell me, ‘I was about to go fight and then I thought about it. Let me sit down and ask them “what’s the problem?” first, before I, I just believe what somebody told.’”—*Participant 2.*

Coaches also noted that they perceived added influence as role models due to being from the same communities and similar backgrounds as the youth. They discussed ways they used their own life experiences to inform how they approached youth going through similar situations. A unique connection in New Orleans was that coaches who had experienced Hurricane Katrina could support youth as they dealt with the effects of Hurricane Ida in Fall 2021.

“That’s why I like even coming to work, for them to see like, someone…who they could probably relate to, like doing something that’s right instead of…because I know when they leave school, it’s easy to see somebody doing something that’s not right.”—*Participant 3.*

“With the hurricane, I just gave them what we did not get as children. So, coming back from school during Hurricane Katrina, we were not asked…how we felt about the situation. But like [after Ida], we sat down and we had a conversation. I did not get that as a child, and I still had to experience all of the hurricanes and just figure it out. But I want them to be not afraid of it.”—*Participant 1.*

### 5.2 Increased opportunity for physical activity facilitated by near-peer relationships

The interview and focus group discussions indicated that near-peer relationships between coaches and youth were also perceived as a catalyst for increased youth participation in sports and physical activity. Coaches talked about tailoring the programming to maximize youth engagement and enjoyment as they built relationships with youth and learned more about their interests. Some coaches shared about starting new sports or activities at their schools that were not previously offered, such as flag football and a majorette program. Coaches also viewed active demonstration of skills or joining in the game alongside the youth as facilitating greater youth excitement for participation in physical activity.

“When we play with them, it gives them a thrill, like oh I’m ‘bout to beat Coach!”—*Participant 4.*

In focus groups, some participants described themselves as initially resistant to being active or “not really a sports person,” but noted they were more willing to participate as the coaches consistently showed up for them and invested in relationships. Similarly, one of the coaches talked about how over time, as he cultivated trust and youth bought into the program, he noticed a transition from youth coming to practice late or unprepared to their being ready to go as soon as he came outside.

“Building a culture was literally the difference between pulling teeth and me walking out there [now], and just my presence is like, oh, is [Coach] here? Hey bro line up.”—*Participant 2.*

At some schools, coaches were also available to offer both a listening ear and a physical activity outlet if a student was struggling during classroom time. Coaches would engage youth in conversation while doing something active and help them get to a place where they were ready to return to class. At two of the schools that participated in this study, coaches had opportunities to work with younger children in addition to the youth who were the focus of the COACHES project. With younger grades, coaches provided structured activities during recess, ensuring this was a time students spent being active, rather than sitting. The relationships with coaches also led youth to seek out coaches for both conversation and physical activity when they had free time.

“When I go in the gym, that’s where I be in when we have free play, me and [Coach] just go and do one versus ones on either side of the court.”—*Participant 15.*

Of note, there were some participants in one of the focus groups, who were negative cases in terms of this subtheme. The negative cases were in the only focus group that included participants who identified as female, and they expressed feeling like the sports offered during programming were more often “boys sports.” They also indicated they wanted their coaches more involved in physical education classes, which they said would motivate them to be more engaged and play harder.

“Yeah, have some fun activities. And activities for girls too, not just basketball and football.”—*Participant 19.*

### 5.3 Limitations to the impact of near-peer relationships

As coaches and youth discussed their experiences, they also identified limits in the extent to which the mentoring relationships and the broader program could impact youth physical activity and holistic health. Coaches highlighted the demanding nature of the work at multiple levels—physically, mentally, and emotionally—and indicated that limited time kept them from investing as deeply as they wished.

“I start feeling as if I’m not giving everyone enough time, but it’s a lot of students and at the end of the day, it is, it’s almost impossible to give every child all the time that they need.”—*Participant 3.*

“Like, they come in with that weight and you take it. Right? And in some situations, you almost feel like because you like their village and they have confided, and trust you so much, you almost feel like you have to kind of help, you know figure something out.”—*Participant 2.*

Both coach interviews and informal interviews with program staff indicated part of this limitation came from constraints in the amount of hours coaches could be offered. Under current funding structures for operating the program, most coaching positions are less than full-time. Coaches described challenges from having to hold multiple jobs in order to meet their financial obligations, and program staff perceived that the limited hours had a negative impact on coach retention.

Beyond the demands on coaches, both youth and coaches identified broader challenges to physical activity and overall wellbeing in the community that the program alone could not address. Participants perceived that the disruptions during the case study period had taken a toll on the community in multiple ways. Coaches and program staff noticed a high frequency of behavioral issues at school, which they attributed to many stresses youth faced in their home environments and changing learning environments. They also described high teacher turnover and challenges with school culture that at times limited program implementation. Youth and coaches alike described barriers they felt to being active and making healthy choices in their communities, specifically highlighting an awareness of high levels of violence.

“So like one of the rules [during virtual instruction was] nobody else could be in your camera. But like if you living with like a large group of family, how can you know, how can you help that? You know, so it’s just like they carried a lot of stuff on them when they came [to school in-person].”—*Participant 2.*

“Well, one thing that’s been kind of stopping me is the violence, stopping me from actually like going a lot of places because like, you never know, like what can happen to you. So I’ve been kind of slacking.”—*Participant 14.*

## 6 Discussion

This case study describes participant perceptions of their experience with a school-based SBYD program during a time period of major disruption to school and programming schedules. In line with other SBYD program research (32, 33), participants identified the coach-youth mentor relationship as a key element of the program experience. In the present study, the similarities in background and relatively small age difference between coaches and youth were specifically highlighted as factors that contributed to close mentorship relationships. Beyond shared demographics, shared experiences due to living in the same community were perceived by coaches and youth as creating opportunity for greater depth of connection that extended beyond the program. Despite acknowledged potential benefits of near-peer relationships in sports and physical activity, recruiting coaches from local communities can be difficult (20). The formation of these relationships and the level of meaning coaches and youth ascribed to them amidst so many challenges to program implementation indicate the importance of SBYD programs continuing to work toward recruiting and retaining near-peer coaches from the communities they serve, despite the difficulties.

In this program, both near-peer relationships and the school-based setting were identified as facilitators of increased opportunities for youth physical activity. Coaches started new programs and incorporated additional time for physical activities at their school sites, and the majority of youth indicated their coaches helped increase their interest in sports. From our observations and informal interviews, the school-based nature of the SBYD programs in New Orleans seemed to be an important factor in maintaining programming in some form despite all the disruptions. Schools had to find ways to continue learning, and thus coaches were still able to connect with youth and offer opportunities for physical activity within alternative models. Community-based programs, in contrast, given that they exist outside the school day, could be canceled or slower to pivot to virtual programming. Our observations are in agreement with previous work indicating the important role of school-based programs in extending physical activity opportunities to all students regardless of their perceived athletic ability or intrinsic interest in sports (34) and the need for programs to offer a range of options that align with the interests and strengths of each particular youth population (35). More research is needed to understand the impact of SBYD programs administered during the school day (36), particularly in regard to youth who are less likely to engage in sports or physical activity on their own.

Although there was a perceived increase in physical activity opportunities for youth at these New Orleans schools, this study also reinforces the challenges that have been identified in quantitatively reporting on physical health outcomes of SBYD programs (19). Since the COVID-19 pandemic, schools have been less willing to participate in external research (37), a reluctance—understandable given the many demands placed on schools—experienced firsthand by our research team. The data collection challenges we faced point to an opportunity for greater collaboration between researchers, program partners, and schools to identify indicators and data collection methods that provide evidence of program efficacy while minimizing burden on schools.

While this study identified a potentially important role for near-peer relationships in promoting youth physical activity and wellbeing, participants recognized limitations in the ability of a single program to address the social issues youth were facing. The need for SBYD programs to expand beyond a focus on individual development toward greater integration into the community has previously been identified (20, 21), and community capacity building has been put forward as a framework for enhancing program impact (38). The Up2Us Sports SBYD program in New Orleans already demonstrates some community capacity building strategies through coach recruitment and school partnerships. Training leaders from within the community can facilitate diffusion of youth development principles through social networks and expand the supportive environment beyond the program setting (38). Furthermore, integration of SBYD into the school setting has been shown to improve school climate perception and school connectedness (36). Continued efforts by SBYD programs to invest in local leadership development and deepen interorganizational collaboration is vital to work toward “changing the odds” rather than helping youth from underserved communities “beat the odds” (38, 39).

## 7 Conceptual and methodological constraints

This case study has several limitations that are important to recognize. First, factors outside our control related to school schedules and coach availability limited the representativeness of the coaches and youth who participated in this study and the depth of insight we were able to gain. With girls already less active than boys on average (1) and predominantly male-identifying voices in this case study, more work is needed to understand the role of near-peer relationships among female-identifying and gender non-conforming youth in SBYD programs (40). We also recognize the potential for selection bias in having coaches recruit youth participants, as those who were more engaged in programming may have been more likely to participate. In the interviews and focus groups, there is also potential for social desirability bias from coaches and youth providing answers they believe facilitators wanted to hear. To minimize social desirability bias, the facilitators endeavored to create a comfortable environment and invited honest feedback at multiple points during the interviews and focus groups. Lastly, the confluence of unique events that affected Up2Us school-based programs in New Orleans over the case study period also limits generalizability to other contexts. Despite these limitations, this case study provides valuable insight into the experiences of youth in urban, under-resourced communities that can inform programs and intervention targeting the multiple facets of youth health.

The combination of near-peer coaches and integration of programming into the school day distinguishes the New Orleans Up2Us SBYD program described in this case study from the majority of SBYD programs that have previously been described. During a series of major external stressors on top of ordinary day-to-day challenges, near-peer relationships still appeared to promote factors associated with positive youth health outcomes, namely physical activity and life skill development. The school-based setting of program delivery allowed for greater continuity in programming relative to out-of-school settings during the intervention period, and this consistency demonstrates one of the benefits of partnerships between SBYD programs and other community entities. Further research into the role of near-peer SBYD coaches in schools is needed to inform best practices to increase youth physical activity and improve both individual and community health outcomes.

## Data availability statement

The raw data supporting the conclusions of this article will be made available by the authors, without undue reservation.

## Ethics statement

The studies involving humans were approved by the George Washington University Committee on Human Research Institutional Review Board. The studies were conducted in accordance with local legislation and institutional requirements. Participants 18 and older provided their written informed consent to participate. For participants under 18, written informed consent was provided by the participants’ legal guardians/next of kin.

## Author contributions

CS: Conceptualization, Formal analysis, Writing – original draft. JM: Conceptualization, Project administration, Writing – review & editing. WG: Conceptualization, Methodology, Writing – review & editing. JS: Conceptualization, Resources, Supervision, Writing – review & editing.
